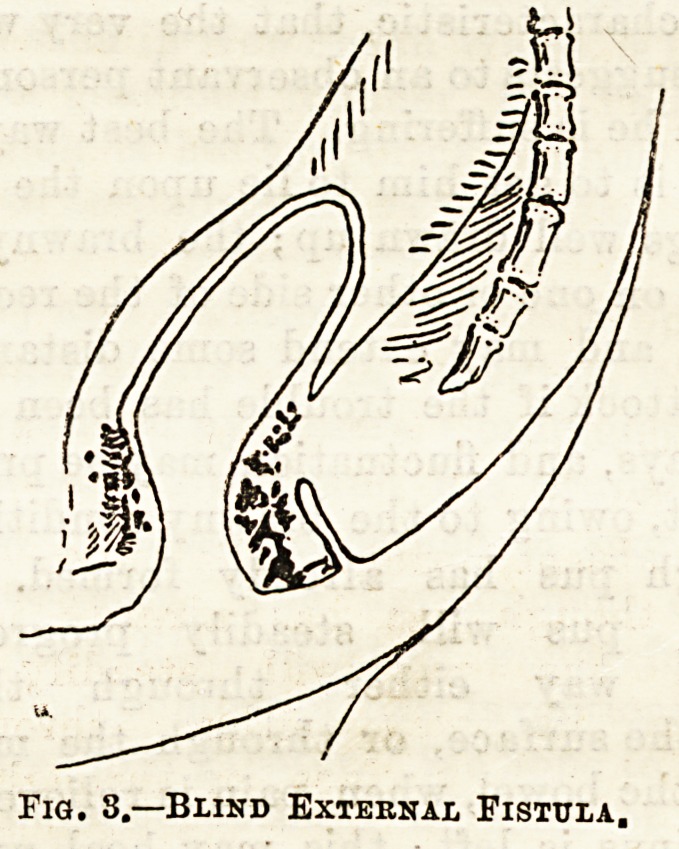# Ischio-Rectal Abscess and Fistula

**Published:** 1895-11-16

**Authors:** E. Percy Paton


### Medical Progress and Hospital Clinics.

[*The Editor will be glad to receive offers of co-operation and contributions from members of the profession. All letters should be addressed to* The Editor, at the Office, 428, Strand, London, W.C.]

### ISCHIO-RECTAL ABSCESS AND FISTULA.

By E. Percy Paton, M.D., M.S., F.R.C.S.

Two forms of ischio-rectal abscess are usually described, which are distinguished from one another, not so much because of any very essential difference as to their results, but because of the marked difference in their symptoms. They are the acute and the chronic.

The symptoms of the former, as the term acute implies, are very rapidly developed. Pain is one of the most prominent troubles, and is the first thing that calls the patient's attention to the fact that anything is amiss. At first it may only be present on defæcation; but soon it becomes continuous, and, in addition, there is marked swelling, redness, and brawniness, with acute tenderness over the affected fossa. This tenderness prevents the patient from being able to sit down without great pain, and when he does sit down, he takes care to do so in such a manner as to rest his weight only on the sound ischial tuberosity. This is so characteristic, that the very way in which he does so, suggests to an observant person the trouble from which he is suffering. The best way to examine the patient is to get him to lie upon the painful side with the legs well drawn up; the brawny œdematous tender area on one or other side of the rectum is then easily seen, and may extend some distance laterally over the buttock if the trouble has been progressing for a few days, and fluctuation may be present, but is often absent, owing to the brawny condition of parts, even though pus has already formed. If left to itself the pus will steadily progress till it makes its way either through the thinned skin on to the surface, or through the mucous membrane into the bowel, when pain is relieved, but a discharging sinus is left; this may heal up, but more often, if it has been thus left to itself, degenerates into a fistula.

The onset and course of the chronic form is much more insidious and slow, and gives rise to such slight symptoms, and to such comparatively little inconvenience, that not infrequently a very considerable collection of pus has formed before much notice is taken of the swelling, which being out of sight the patient does not so easily observe. When this has happened a very considerable denudation of the rectal canal from the surrounding tissues sometimes takes place, so that the pus has spread from one ischio-rectal fossa behind (rarely in front) of the rectum into that of the opposite side, giving rise to what is known as the horseshoe abscess. It may also spread widely out on to the buttock, so that a bad untreated case may give rise to a very considerable amount of riddling of the surrounding parts with sinuses.

**Causes.**—These are various, but no doubt some cases arise by direct inoculation of the ischio-rectal fat, which has but a feeble vitality, with septic material by puncture of the rectum by such a foreign body as a fish bone. Or abscess may occur from absorption from a fissure about the anus or by suppuration of a thrombosed pile. These causes generally give rise to the acute form of inflammation. The chronic form is usually found in patients with broken-down constitutions and in general poor health, especially patients suffering from tubercle in the lungs, and no doubt in not a few of these cases the abscess itself is tubercular, though the exciting cause may have been sitting on a cold damp seat, or some other slight traumatism, &c.

**Treatment.**—The indication is to let out the pus at the earliest possible opportunity, and then, if possible, to prevent the formation of a fistula, so that as soon as brawny infiltration and œdema are present, fluctuation should never be waited for, as it is often absent when there is plenty of pus. The best thing, no doubt, is, following Allingham, to give an anæsthetic, as then the abscess can be thoroughly treated in a manner which would otherwise be much too painful. The patient is placed on the diseased side, with the legs well drawn up, and a free incision made into the abscess either radiating from or skirting the anus, the collection of fluid being made more obvious if necessary by inserting the left finger into the rectum and pressing it outwards. The pus which escapes is always very foul smelling, even though there may be no direct communication with the bowel. This is almost always the case with any collection of fluid in close proximity to the alimentary tract. A finger is now passed into the abscess cavity, which is carefully explored, and if any pockets are found, the partitions between these are broken down and the cavity carefully washed out with 1 in 40 carbolic; it is then filled with a strip of lint, either soaked in carbolic oil or smeared with vaseline, care being taken not to distend the cavity, but to put in the lint in such a way that the side walls of the abscess can only come in contact at its very bottom.

Fig. 1.—Complete Fistula.

Allingham advises that this plug should be kept in for three days, while in the meantime the bowels are kept confined by opium, and the patient kept at rest, at any rate, on the sofa, if not in bed. After the first plug has been removed, the cavity must still be compelled to fill up from the bottom, by keeping the sides apart with a small plug of lint or a little cotton wool. It should not, however, be stuffed so as to distend it, as this is not unlikely to keep open the sinus instead of promoting its closure. It is only by careful attention to details that these abscesses can be prevented

Fig. 2.—Blind Internal Fistula.

from giving rise to that common sequela, fistula in ano.

This result—namely, fistula in ano—is so common that in order to complete the description of the results of ischio-rectal abscesses, it must here be briefly dealt with. Three varieties are distinguished from one another—namely, the complete, the blind internal, and the blind external.

In the complete fistula (see fig. 1), as its name implies, there is a complete channel from the cutaneous surface through into the bowel. The internal opening is most commonly situated between the internal (or involuntary) and the external (or voluntary) sphincters, or it may be above both sphincters, or superficial to both, the former of these two being very uncommon, and the latter is only the result of a superficial or anal abscess.

The blind internal fistula (see fig. 2), is one in which the sinus opens into the rectum, and has no external opening, while the blind external (see fig. 3), has only an opening through the skin, and no communication with the bowel.

The question here may well be asked why is it that these sinuses when once fully established and then left untreated, or even when treated by anything short of operation, so rarely close up? There are no doubt several reasons. In those cases in which an opening into the bowel is present, it is very obvious that the frequent entrance of fæcal matter will be a constant bar to sound healing, while in all varieties the constant disturbance to which the parts are liable, and the comparatively low vitality of the tissues in this region, tend towards the same result. Then, again, those which show the least inclination to heal, at any rate of the blind external variety, are those in which there is present some constitutional weakness, and should this postpone healing for some time, the sinus has then got firmly established, with hard, sometimes almost cartilaginous, walls, and so will not close up.

The symptoms to which a fistula may give rise are often very slight, consisting merely of a slight semi-purulent watery discharge, and in the blind internal variety of a purulent discharge which comes from the anus and occasions pain on defæcation. In many cases the most troublesome thing is the recurrence of attacks either acute or subacute of the ischio-rectal abscess, which gave rise to the sinus in the first instance, and with each of these attacks the sinus very often spreads

Fig. 3.—Blind External Fistula.

in various directions into the buttock or up the side of the rectum, thus diminishing the possibility of spontaneous closure, and increasing the extent of the operative interference necessary for its treatment.

## Figures and Tables

**Fig. 1. f1:**
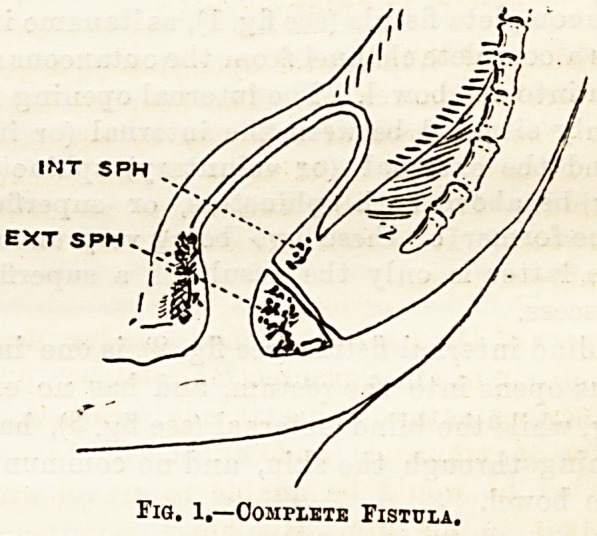


**Fig. 2. f2:**
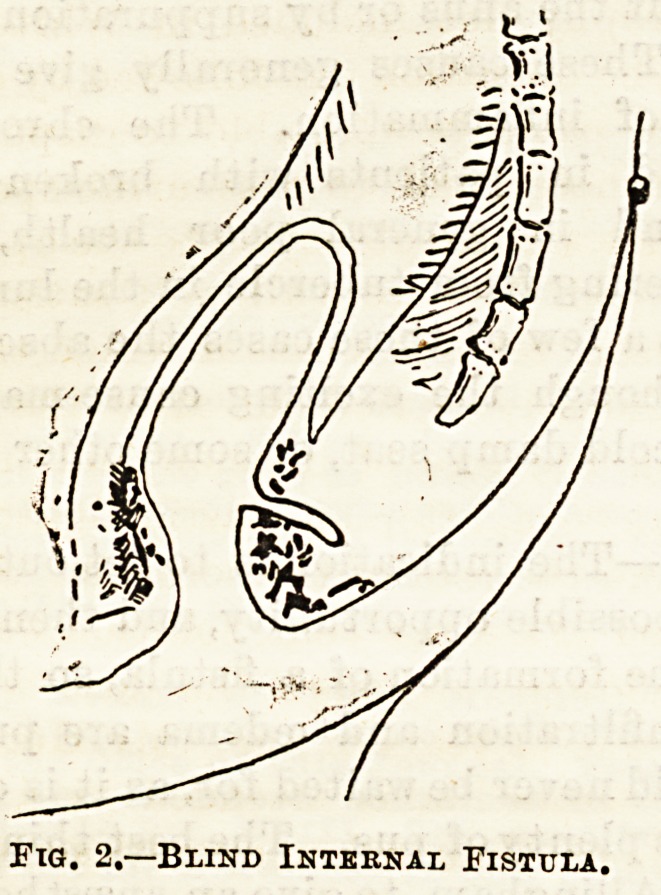


**Fig. 3. f3:**